# Highly purified extracellular vesicles from human cardiomyocytes demonstrate preferential uptake by human endothelial cells

**DOI:** 10.1039/d0nr04278a

**Published:** 2020-09-24

**Authors:** Limor Zwi-Dantsis, Charles W. Winter, Ulrike Kauscher, Arianna Ferrini, Brian Wang, Thomas E. Whittaker, Steve R. Hood, Cesare M. Terracciano, Molly M. Stevens

**Affiliations:** a Department of Materials Department of Bioengineering, Institute for Biomedical Engineering Imperial College London, London, UK; b National Heart & Lung Institute, Imperial College London, London, UK; c Medicines Research Centre, GlaxoSmithKline Research and Development, Stevenage, UK

## Abstract

Extracellular vesicles (EVs) represent a promising cell-free alternative for treatment of cardiovascular diseases. Nevertheless, the lack of standardised and reproducible isolation methods capable of recovering pure, intact EVs presents a significant obstacle. Additionally, there is significant interest in investigating the interactions of EVs with different cardiac cell types. Here we established a robust technique for the production and isolation of EVs harvested from an enriched (>97% purity) population of human induced pluripotent stem cell (iPSC)-derived cardiomyocytes (CMs) with size exclusion chromatography. Utilizing an advanced fluorescence labelling strategy, we then investigated the interplay of the CM-EVs with the three major cellular components of the myocardium (fibroblasts, cardiomyocytes and endothelial cells) and identified that cardiac endothelial cells show preferential uptake of these EVs. Overall, our findings provide a great opportunity to overcome the translational hurdles associated with the isolation of intact, non-aggregated human iPSC-CM EVs at high purity. Furthermore, understanding in detail the interaction of the secreted EVs with their surrounding cells in the heart may open promising new avenues in the field of EV engineering for targeted delivery in cardiac regeneration.

## Introduction

Extracellular vesicles (EVs) have gained much attention due to their reported roles in delivering protective and regenerative biomolecules to aid the repair of tissue damage.^1–3^ “EV” refers to a broad collection of cell-secreted vesicles comprising exosomes and microvesicles.^4,5^ Exosomes tend to be smaller (50–150 nm diameter) and assemble within the endosomal compartment of the cell. They are released from cell membranes after multi-vesicular body fusion with the cell plasma membrane.^5,6^ Microvesicles, on the other hand, bud directly from the plasma membrane and tend to be slightly larger, ranging between 50 to 500 nm.^6^ To date, separation techniques cannot completely distinguish the two populations due to the overlap in their physical and biochemical characteristics.^5,7^ EVs are secreted from different cell types and can be isolated from serum-free cell culture media *in vitro* as well as from various bodily fluids.^7^ They have a broad molecular composition, which is reflective of their cellular origins, carrying a complex assortment of lipids, proteins, nucleic acids and transcriptional messengers (mRNAs and microRNAs) that can be transferred to recipient cells to mediate intracellular communication.^8–10^

Recently, EVs have been leveraged as a new cell-free therapeutic for treating cardiovascular diseases, which are major contributors to morbidity and mortality globally.^11^ So far, strategies to restore cardiac function have mainly focused on stem cell-based therapies. The reprogramming of autologous somatic cells into induced pluripotent stem cells (iPSCs) has provided a key translational technology to derive highly pure and functioning cardiomyocytes to replenish the injured heart through cellular engraftment.^12,13^ However, limited cell targeting to the infarct area combined with the challenge of controlling cell fate and viability after ischemic insult remains a major challenge.^14^ More recently, instead of delivering cells systemically or by intramyocardial injection, iPSC-derived cardiomyocytes, and other stem cells have been localized to damaged regions within biomaterial patches.^15–17^ Nevertheless, poor integration between the biomaterial and pre-existing cardiac tissue has raised safety concerns and limited the translatability of these approaches.

The beneficial therapeutic properties which are attributed to cell therapies for cardiac regeneration are now widely regarded to be driven by cell secretion of paracrine signalling factors, of which EVs have emerged as a key component.^18^ EVs secreted from various cell types have been shown to improve heart function,^19^ enhance angiogenesis,^20^ and decrease fibrosis21 in several animal models of myocardial infarction. For example, EVs harvested from conditioned media of mesenchymal stem cells (MSCs) were reported to have cardio-protective activity in a rodent model of myocardial ischemia-reperfusion injury, decreasing infarct size and maintaining cardiac performance compared to untreated controls.^22^ Similarly, EVs isolated from cardiosphere-derived cells (CDCs) and human embryonic stem cell-derived cardiovascular progenitors (hESC-Pg) exhibited cardioprotective effects in animal models of myocardial infarction.^1,23^ Recently, the therapeutic potential of EVs derived from human umbilical cord MSCs and EVs derived from human iPSC derived cardiomyocytes (CMs) on injured hearts was further enhanced by encapsulating these EVs within hydrogels.^24,25^ Although the results of these studies are very promising, EVs used for cardiac repair have mostly been isolated using ultracentrifugation (UC) or polymer mediated precipitation.^18,26^ Recent advances in EV isolation including size exclusion chromatography (SEC),^27^ tangential flow filtration,^28^ field flow fractionation29 and acoustic separation. 30 Novel microfluidic methods have also been developed to enable isolation of EVs with high efficiency and purity in small sample volumes.^31,32^ These approaches may shed new light on the properties of cardiac EVs for future therapeutic applications.

**Fig 1:**
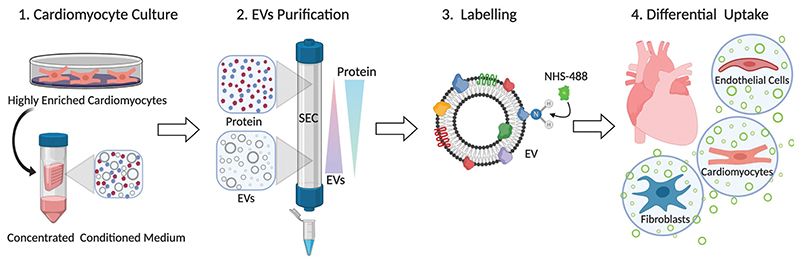
Schematic illustration of workflow. Enriched human CMs (>97%) were differentiated from human iPSC in glucose-depleted media supplemented with lactate. EVs and soluble cellular proteins secreted into the serum-free media were collected and concentrated. To purify EVs from soluble protein contaminants, size-exclusion chromatography (SEC) was performed and highly pure CM-EVs were obtained. The purified EVs were fluorescently labelled using an amine reactive fluorescent dye. This method enabled quantitative assessment of EVs interactions with different human cellular components of the myocardium (endothelial cells, cardiomyocytes and fibroblasts).

We demonstrate here the production and isolation of high purity EVs from a homogenous population of human iPSC-CMs using SEC. Our methodology seeks to complement others work in EV isolation and provide the first report of SEC isolation of EVs from human iPSC-CM, to the best of our knowledge. We then developed a fluorescent labelling strategy for EVs that enabled us to track and quantify their cellular uptake by three of the major cellular components of the myocardium, namely fibroblasts, cardiomyocytes and endothelial cells (Fig. 1). Our findings provide timely insights regarding the pharmacodynamic interactions of cardiac EVs that may pave the way for the development of new nanoscale cell-free targeting therapies in cardiac regeneration.

## Materials and methods

### Cell culture

An undifferentiated human iPSC line (Thermo Fisher Scientific) was routinely maintained in complete Essential 8 (E8) cell culture medium (Thermo Fisher Scientific) on 6-well plates coated with 1 : 100 growth factor–reduced Matrigel (R&D Systems). The cells were passaged every 3–4 days using 0.5 mM EDTA in D-PBS (Thermo Fisher Scientific) and re-plated in E8 medium supplemented with 2 μM of the ROCK inhibitor Thiazovivin (Stratech Scientific) for the first 24 h following passaging. Human cardiac microvascular endothelial cells (hCMEC; PromoCell) were grown in PromoCell Cell Growth Medium and passaged using PromoCell DetachKit. The media was changed every two to three days. Human cardiac fibroblasts (hCFib; kindly provided by Prof. Terracciano) were cultured in DMEM high glucose (Thermo Fisher Scientific) supplement with 10% (v/v) FBS (Thermo Fisher Scientific) and dissociated using 0.25% trypsin (Sigma-Aldrich).

### Cardiomyocytes differentiation

To induce cardiomyocyte differentiation, two types of serumfree differentiation media were used: (1) CDM3, consisting of RPMI-1640 (Thermo Fisher Scientific), 213 μg mL^−1^ L-ascorbic acid 2-phosphate (Sigma-Aldrich), 500 μg mL^−1^ recombinant human serum albumin (Sigma-Aldrich), and 1% (v/v) penicillin/ streptomycin (100 U mL^−1^ and 100 g mL^−1^, respectively; Thermo Scientific); or (2) B27, containing RPMI-1640 supplement with 2% B27 (v/v) (Thermo Fisher Scientific) and 1% (v/v) penicillin/streptomycin. When the human iPSCs reached 80–90% confluence, the differentiation was started by changing the E8 culture medium to B27 (minus insulin) or CDM3 supplemented with 6 μM CHIR99021 (tebu-bio) for one or two days, respectively. On day three, medium was replaced with CDM3 or B27 (minus insulin) supplemented with 2 μM Wnt-C59 (Stratech Scientific) for two additional days. From day 5 onwards, the cells were cultured with CDM3 or B27 media, and medium was changed every other day. On day 8–10, spontaneous contraction could be identified. To further increase cardiomyocyte purity, the differentiated cells were subjected to glucose starvation on days 10–16 post-differentiation. At day 10, the medium was changed to LAC medium, composed of glucose-free RPMI-1640 (Thermo Fisher Scientific) supplemented with 5 mM sodium DL-lactate (Sigma-Aldrich) and 1% (v/v) penicillin/streptomycin for 3 days. At day 13, cells were returned to B27 or CDM3 differentiation media for 24 h. At day 14, the medium was changed back to LAC medium for a second glucose deprivation cycle for an additional 3 days.

### EV isolation

At day 17 of differentiation, conditioned media was collected and centrifugated at 400*g* for 5 min to remove cells, debris and apoptotic bodies. The resulting supernatant was filtered through 0.45 μm pore size membrane filters (Merck), stored at −80°C and later thawed on ice, or immediately concentrated through Amicon Ultra Centrifugal filter units (MWCO = 100 kDa; Merck) to a final volume of 500 μL by repeated centrifugation at 5000g. A 30 cm glass chromatography column (Lenz Borosilicate) with an internal diameter 1 cm was packed using 30 mL of Sepharose CL-2B (Sigma-Aldrich) to provide a total gel length of 24 cm. After washing the column with D-PBS, the concentrated conditioned media was loaded and eluted with D-PBS, and 1 mL fractions were collected. For the cellular studies and cryo-TEM imaging, the EV rich, proteinlow fractions (8−12) were pooled and concentrated to ≤600 μL on an Amicon Ultra-4 Centrifugal Filter Unit (MWCO = 100 kDa; Merck).

### Flow cytometry

For LAC-CM-EVs uptake evaluation, the cells were incubated with 10^10^ particles per mL of LAC-CM-EVs overnight. The next day, the CMs were dissociated into single cells with TrypLE (Thermo Fisher Scientific), while hCMEC and hCFib were dissociated using PromoCell DetachKit and 0.25% trypsin, respectively. Subsequently, the cells were incubated with a fixable viability dye eFluor 450 (eBiosciences), washed in D-PBS, filtered using 40 μm cell strainer (Corning) and fixated using 4% (v/v) paraformaldehyde (Sigma-Aldrich) in D-PBS. For analysing intracellular cardiac troponin T (cTnT) expression, the CMs were further permeabilized using the FIX & PERM kit (Thermo Fisher Scientific) and stained with a primary antibody mouse anti-human cardiac troponin T (1 : 100; R&D systems), followed by a secondary antibody goat anti-mouse IgG^2a^ Alexa Fluor 488 (1 : 200; Thermo Fisher Scientific) at 4 °C. Analytical flow cytometry was performed using LSR Fortessa flow cytometer (BD Biosciences), and analysis was carried out with FlowJo V10 software.

### Immunostaining studies

Dissociated cells were fixed with 4% (v/v) paraformaldehyde, rinsed three times in D-PBS and blocked with 5% (v/v) horse serum (Sigma-Aldrich). CMs and hCMECs were incubated overnight at 4 °C with primary antibodies against sarcomeric α-actinin (1 : 200; Sigma-Aldrich) and CD31 (1 : 200; Thermo Fisher Scientific), respectively. The following day, the preparations were rinsed three times in D-PBS and incubated with secondary antibodies: donkey anti-mouse IgG (Stratech Scientific) or goat anti-mouse IgG^2a^ (Thermo Fisher Scientific) at a dilution of 1 : 200. hCFib were stained with Alexa Fluor 555-phalloidin (Thermo Fisher Scientific), a peptide that binds to and label actin in cells. All samples were then washed in D-PBS, mounted with Vectashield Antifade Mounting Medium (Vector Laboratories) and nuclei were counterstained with DAPI (1 : 1000; Sigma-Aldrich). The specimens were examined with a laser scanning confocal microscope (Leica SP8).

### Nanoparticle tracking analysis (NTA)

**Fig. 2:**
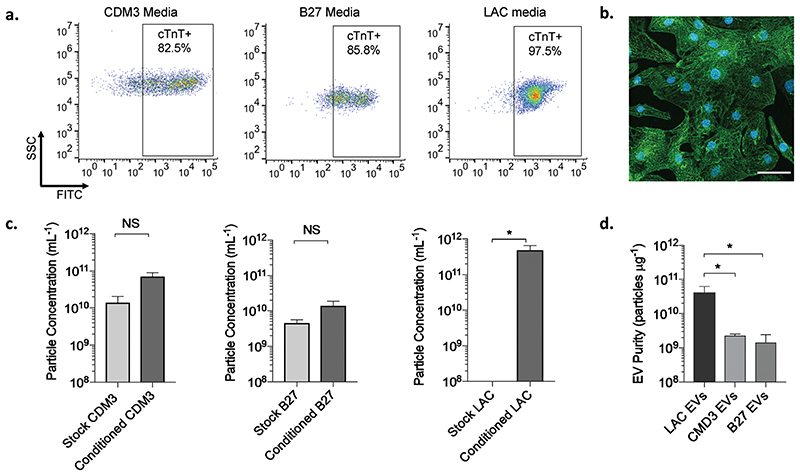
CM differentiation capacity and purity of isolated EVs in different media conditions. (a) Representative dot plots of side scatter (SSC) versus FITC fluorescence intensity of cardiac troponin T positive (cTnT^+^) cells after differentiation under different media conditions: CDM3 media (left), B27 media (middle) and LAC media (right). (b) A representative image of immunostaining for sarcomeric α-actinin (green) shows highly enriched population of human CMs obtained after culturing in glucose-depleted media supplemented with lactate (LAC media). Nuclei were counterstained with DAPI (blue). Scale bar, 50 μm. (c) Particle concentration (mean + s.e.m.; *N* = 3–7; unpaired Welch’s *t*-test) for stock and conditioned media from CDM3 media (left), B27 media (middle) and LAC media (right). (d) Purity comparison for EVs (mean + s.d.; *N* = 3–7; one-way ANOVA) purified from the different types of cell culture media based on the particle to protein ratio. * p < 0.05; NS: non-significant; N: independent replicates.

**Fig. 3:**
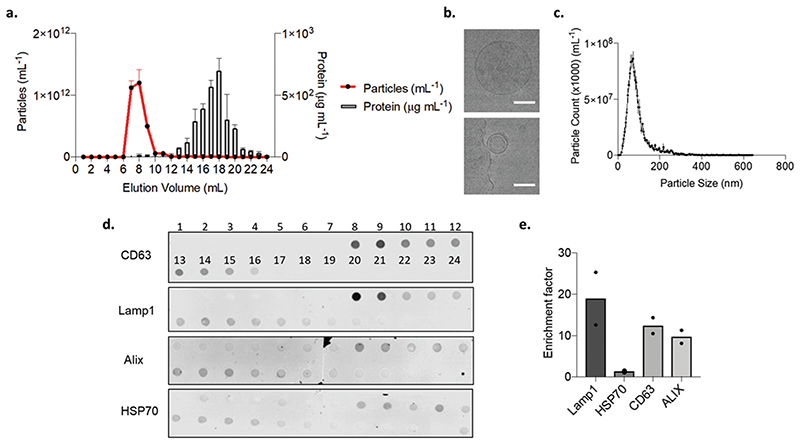
LAC-CM-EVs purification and characterization. (a) Particle concentration (mean ± s.d.; *n* = 5) and total protein content (mean + s.d.; *n* = 3) of 1 mL column fractions eluting from a 24 cm SEC column of concentrated LAC conditioned media. (b) Representative cryo-transmission electron microscopy (cryo-TEM) images of single purified LAC-CM-EVs. Scale bar, 200 nm. (c) A representative particle size distribution profile from nanoparticle tracking analysis (NTA) of EV rich fractions (fraction 8). (d) Dot blot image for qualitative evaluation of the elution of endosomal (CD63 and Lamp1) and cytoplasmic (Alix and HSP70) proteins. (e) Quantitative evaluation of dot blot for protein enrichment factor from mean signal intensity in EV rich fractions (8 to 9) compared to mean soluble protein rich fractions (15 to 24). Data presented as individual points and mean (*N* = 2). n: technical replicates; *N*: independent replicates.

**Fig. 4:**
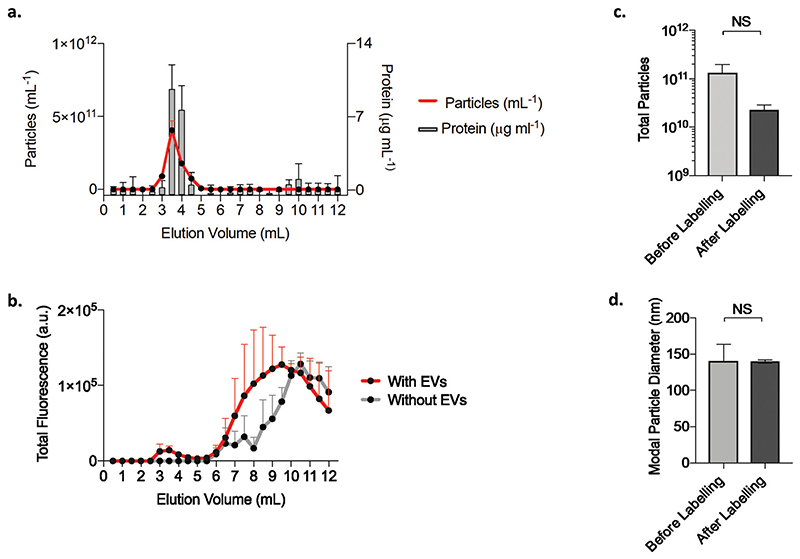
EVs fluorescence labelling. (a) Particle concentration (mean + s.d.; *n* = 5) and total protein content (mean + s.d., *n* = 9) for each of the 0.5 mL volume fractions eluting from the qEV column of unlabeled LAC-CM-EVs. (b) qEV elution profile for the total fluorescence intensity of fluorescently labelled LAC-CM-EVs and control of labelling dye only without EVs (mean + s.d., *N* = 2). (c) Total particle count for LAC-CM-EVs before and after fluorescence labelling (mean + s.e.m., *N* = 3; *p* = 0.23; unpaired Welch’s *t*-test). (d) Particle size of LAC-CM-EVs before and after labelling (mode size + s.e.m., *N* = 3; *p* = 0.97; unpaired Welch’s *t*-test). NS: non-significant; n: technical replicates; N: independent replicates.

To determine particle size and concentration, NTA was performed using a NanoSight NS300 instrument (Malvern Ltd) equipped with NTA 3.2 analytical software. Samples were first thawed on ice and diluted in D-PBS to achieve a count of between 10^8^ and 10^9^ particles per mL. Each sample was then loaded in the sample chamber and the camera focus was adjusted. A camera level of 14 (Fig. 4) or 15 (Fig. 2 and 3) was used for the recordings and the detection threshold was fixed at 5. All comparisons were made at consistent camera levels. Five 30 or 60 s videos were recorded for each sample. Batch process function was applied to analyze the measurements and the results were exported to Microsoft Excel for further analysis.

### Dot blot

To elucidate the presence of EV-associated protein markers within the SEC fractions, dot blots were conducted using a BioDot apparatus in accordance with the manufacturer’s directions (BioRad Laboratories). For each 1 mL column fraction, 250 μL was loaded per well. The sample was allowed to flow under gravity onto the TBS-soaked nitrocellulose membranes, pore size 0.45 μm (BioRad Laboratories). To minimize non-specific antibody adsorption, the nitrocellulose membranes were incubated for 1 h in 5% (w/v) non-fat dry milk (Sigma) in TBS-T (BioRad Laboratories). Subsequently, the nitrocellulose membranes were incubated overnight at 4 °C with the desired primary antibody diluted at 1 : 1000 in TBS-T, including CD63 (Thermofisher), Lamp1 (Cell Signaling Technologies), HSP70 (New England Biolabs) and Alix (New England Biolabs). Three TBS-T washes of 10 min were then performed to remove non-specifically adsorbed primary antibodies. The membranes were incubated with near-infrared emitting secondary antibodies (IR-Dyes 680, LICOR). These secondary antibodies were diluted 1 : 10 000 in TBS-T and incubated with the nitrocellulose membranes for 1 h at room temperature. After three further 10 min washes in TBS-T, the nitrocellulose membranes were imaged using the LICOR Odyssey platform. The near infrared emission from the blot was recorded in the 700 nm channel. Signal intensity measurement were quantified using the Image Studio software. Analysis of the enrichment factor for a specific protein in the EV fractions was calculated by dividing mean signal intensity from fractions 8–9 by the mean signal intensity values between fractions 15–24.

### Protein quantification

To determine the total protein concentration, a micro-BCA kit (Thermo Fisher Scientific) was used in accordance with manufacturer instructions. A calibration curve of serially diluted bovine serum albumin (BSA) standards in D-PBS was recorded for their total absorbance at 562 nm using Envision multimodal plate-reader (PerkinElmer). EV samples were diluted in D-PBS to be within the dynamic absorbance range of the calibration curve. Baseline corrected absorbance measurements for the EV samples were substituted into fitted calibration curve equation and this was used to quantify the total protein concentration.

### Cryo-TEM

Holey Carbon Grids (HC200-Cu, Electron Microscopy Sciences) were plasma treated on a Gatan Solaris Plasma Cleaner (conditions: 15 s, O_2_/H_2_). Samples were plunge frozen using a Leica EM GP automatic plunge freezer with the following method: a plasma cleaned grid was loaded into the environmental chamber of the plunge freezer (relative humidity: 90%, temperature: 20 °C) and 4 μL of sample was added onto the carbon coated side. Excess sample was blotted off using filter paper mounted in the environmental chamber and the obtained film was vitrified in liquid ethane. Samples were then stored under liquid nitrogen until imaged. Samples were imaged using a JEOL 2100 plus with 200 kV and the Minimum Dose System. Imaging temperature was −170 °C in a Gatan914 cryoholder. Micrographs were taken using a Gatan Prius SC1000 camera at a magnification of 30,000×.

### Fluorescence labelling of the EVs

To conduct the EV labelling reaction, 2 × 10^11^ purified LAC-CM-EVs were diluted into a total volume of 500 μL PBS. The EV solution was then mixed with 5 μL of 10 mg mL^−1^ NHS-Alexa Fluor 488 (Molecular Probes, Thermo Fisher Scientific) to ensure a homogeneous dye distribution. The labelling reaction was allowed to proceed for 12 h at 4 °C. To purify LAC-CM-EVs, qEV columns (Izon Sciences) were used, with elution profiles being characterised for the (1) unlabelled EVs, (2) labelled LAC-CM-EVs and (3) free labelling buffer. Each 500 μL eluting from the qEV column was collected and assayed for total fluorescence intensity (Enspire, PerkinElmer), protein content was assayed by Micro-BCA assay (Thermo Fisher Scientific) and particle concentration was assessed using NTA as described previously (Nanosight NS300, Malvern Instruments).

### Statistical analysis

**Fig. 5:**
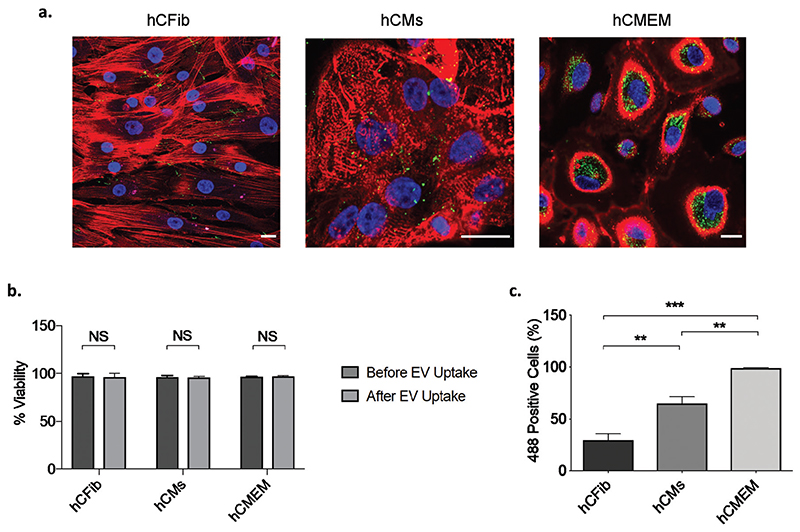
Evaluation of LAC-CM-EV cellular uptake by immustaining and flow cytometry analysis. (a) Internalization of 488-labeled LAC-CM-EVs (green, 1010 particles per mL) 24 h after incubation. hCFib stained for actin with Alexa Fluor 555 phalloidin (red, left); hCMs stained with the cardiac marker sarcomeric α-actinin (red, middle); and hCMEM stained with anti-CD31 (red, right). Nuclei were counterstained with DAPI (blue). Scale bar, 20 μm. (b) Quantitative assessment of the cell viability before and after LAC-CM-EV uptake using flow cytometry. Data shown as mean + s.d. *N* = 3–4 independent experiments; unpaired Welch’s *t*-test; NS: non-significant. (c) Percentage of 488 positive cells reveals differential uptake capacity of the LAC-CM-EVs with elevated uptake observed by the endothelial cells (>98%). Data shown as mean + s.d. *N* = 3–4 independent experiments; one-way ANOVA. **p < 0.01, ***p < 0.001.

Statistical analysis was performed using Prism software (GraphPad). Repeated-measurements one-way ANOVA followed by Tukey post-hoc multiple-comparison analysis was carried out for assessing EV purity (Fig. 2d) and cellular uptake (Fig. 5c). Unpaired Welch’s *t*-tests were used to compare particle concentration (Fig. 2c), total amount of particles (Fig. 4c), particle size (Fig. 4d) and cell viability (Fig. 5b). NTA data measurements are recorded as mean ± standard error (s.e.) while all other measurements are recorded as mean ± standard deviation (s.d.). p values < 0.05 were considered statistically significant.

## Results and discussion

### Isolation of EVs from human CMs cultured in different media conditions

Human iPSCs are a viable source of cells that can be expanded and differentiated into most cell types, including CMs.^33^ Here, we obtained human CMs using monolayer-directed differentiation systems of human iPSC which are the most efficient and scalable approaches currently available.^34^ We then isolated the CM-EVs secreted at day 17 of differentiation from each culturing condition and compared their purity. For that purpose, the CMs were cultured with three types of serum-free media: CDM3 (RPMI supplement with human albumin and ascorbic acid), B27 (RPMI supplemented with B27); or alternatively, at day 10 of differentiation, the cells were exposed to an additional purification step by culturing them in glucose-free LAC media (RPMI without glucose supplement and with lactate). The latter treatment enabled us to eliminate non-CMs that could not survive in a low-glucose environment, yielding a high purity population of CMs that can utilize lactate as their primary energy source.^35^ Flow cytometry analysis of cardiac troponin-positive cardiomyocytes (cTnT^+^) revealed that an enriched cardiomyocyte population (>97% CMs) was obtained when culturing the cells in LAC medium compared to the other two media (82.5% and 85.8% for CDM3 and B27, respectively; Fig. 2a), in agreement with previous studies.^36–38^ The highly homogenous CM population after culturing with LAC media was corroborated by confocal microscopy (Fig. 2b). Cell staining for the sarcomeric protein α-actinin revealed a typical striated pattern which supported successful purification of the CMs following glucose starvation.

To date, UC has been the most popular method for isolating EVs in the cardiovascular field.^39^ Here we offer an alternative method to effectively separate human iPSC-CM-EVs from non-vesicular components using SEC. This methodology has been employed to isolate EVs from MSC,^40^ CPC^41^ and iPSCderived neurons,^42^ among others. However, to the best of our knowledge, SEC has not been reported for purifying EVs from human iPSC-CMs. This powerful chromatographic approach enables simple, efficient and rapid separation of molecules based on their hydrodynamic radius with high reproducibility.^43^ To this end, Nanoparticle Tracking Analysis (NTA) was employed to determine particle concentration of fractions eluting from the SEC column from all three tested stock media (without cells) and from conditioned media after culturing with the CMs. In both CDM3 and B27 media we could detect particles associated with the stock media, while for the LAC medium, the particle concentration fell below the accurate detection threshold (Fig. 2c). This implied that the CDM3 and B27 stock media contained materials that would contaminate EV-containing fractions. Nevertheless, the number of particles isolated from LAC conditioned media was significantly higher than the stock media, indicating that EVs were secreted by the CMs, in accordance with previous reports which support that EVs are released by all cardiac cells and play an important role in the cardiovascular system.^44,45^

To quantitatively assess the purity of the EVs isolated for each of the media conditions tested, the particle to protein ratios were calculated. Webber and Clayton previously devised an EV purity rating scale based on the particle to protein ratio. EV preparations which contained more than 3 × 10^10^ particles per μg of protein were classified as highly pure.^46^ Notably, in our study, LAC-CM-EVs demonstrated superior purity, with a ratio of 4.2 × 10^10^ ± 8.0 × 10^9^ particles per μg protein, which was significantly higher than either CDM3-CM-EVs (2.3 × 10^9^ ± 1.8 × 10^8^ particles per μg) or B27-CM-EVs (1.4 × 10^9^ ± 5.9 × 10^8^ particle per μg; Fig. 2d). Overall, culturing CMs in glucosedepletion media resulted in more homogeneous CM populations and allowed isolation of EVs with higher particle/protein ratios that were consistent with high purity. Deriving these highly purified EVs from human CMs may be critical for future clinical and translational cell-free applications as well as for basic cardiac research.

### Characterization of LAC-CM-EVs

Due to the high purity of the LAC-CM-EVs, we continued to further characterize their properties in terms of purification yield, morphology, size distribution, and the presence of EV-related protein markers. Recently, SEC has been shown to efficiently remove contaminating non-vesicular proteins, plasma proteins and high-density lipoproteins (HDL) from EVs.^47,48^ Here, the purification of concentrated LAC conditioned media was performed using a 1 cm internal diameter SEC column packed with Sepharose CL-2b to a total length of 24 cm, which is substantially longer than previously reported in the literature,^49,50^ and could clearly separate cardiac EVs from soluble protein contaminants for high-quality purification (Fig. 3a). This purification strategy resulted in most EVs and their associated protein eluting between 7–10 mL, since this elution volume contained the highest abundance of particles. By contrast, most protein was detected separately to the EVs and eluted between 15–24 mL. Thus, we demonstrate that SEC is capable of efficiently separating LAC-CM-EVs which have large hydrodynamic radius from other small contaminants such as non-EV proteins in well-defined separation times and with a high degree of reproducibility and sensitivity. Furthermore, for 400 mL of conditioned media collected, it was possible to purify a total of 4 mL of EVs with an average particle concentration of 7.2 × 10^11^ ± 4.0 × 10^10^ particles per mL. Overall, we identified that SEC purification recovered 58% of the EVs initially loaded onto the column which is comparable to other previous reports.^47^ Further improvements to the recovery may be possible by further optimizing the column height, column diameter and sample volume.

To further evaluate the contents of concentrated fractions 7–12 mL, cryo-transmission electron microscopy (cryo-TEM) was performed. This imaging method preserves vesicle membranes in their natural hydrated state and enables direct visualization of LAC-CM-EV morphology. Concentrated LAC-CM-EVs were found to possess a circular shape with unilamellar membrane structure, as shown in Fig. 3b. Their average diameters agree with previous literature reports,^51^ and closely corresponds to the mean particle size recorded in our NTA 126.8 ± 4.7 nm (Fig. 3c). Notably, there was no evidence of structural deformation, aggregation or breakdown as expected with the SEC isolation method, in which the vesicles are subjected to gravity flow rather than the shearing forces applied in UC at high speed.^43^

Next, the total protein and dot-blot analyses were conducted on the first 24 fractions (1 mL each) obtained from the SEC column. We demonstrated that different SEC fractions carried different concentrations of the various endosomal and cytoplasmic protein markers CD63, Lamp1, Alix and HSP70 (Fig. 3d).^52^ We then quantified an enrichment factor between peak EV rich fractions (fractions 8 and 9) and compared this to the mean soluble protein rich fractions (fractions 15–24). We identified that the endosomal protein markers Lamp1 and CD63 were substantially more highly enriched in the EV-containing fractions; while the cytoplasmic proteins Alix and HSP70 showed some enrichment in LAC-CM-EVs, but to a lesser extent than the endosomal protein markers (Fig. 3e). Collectively, our data indicate that EVs released from CMs into LAC conditioned medium were effectively purified from contaminants using the SEC method. We observed that the purified EVs continue to retain their integrity and carry EVs associated transmembrane protein markers. Importantly, SEC separation is advantageous since it is simple, rapid, requires no specialized equipment and poses minimal risk of causing damage or aggregation of the EVs, making this approach appropriate for isolating cardiac EVs for translational research.

### Fluorescent Labelling of LAC-CM-EVs

Next, we developed a simple and highly effective strategy for fluorescently tagging SEC purified LAC-CM-EVs using an N-hydroxysuccinimide ester (NHS) Alexa-488. In previous reports, this labelling method has been applied to ultracentrifugation-purified EVs.^53^ However, inadvertent labelling of non-EV contaminants often present in such samples may obscure EV labelling efficiency. Furthermore, in other uptake studies EVs were typically labelled with non-specific non-covalent membrane dyes^24^ that can potentially also label the cells themselves and give false-positive results;_54_ in our approach the dye is covalently attached to the EV membrane and the free dye is subsequently removed, which enables a better and more reliable approach for tracking EVs within recipient cells. To separate labelled EVs from excess free dye a shorter qEV column was used for purification to minimize sample dilution. Fig. 4a depicts a typical qEV elution profile generated by a sample of purified unlabeled EVs. EVs eluted at the highest concentrations in fractions 3–4 mL, as demonstrated by the increased abundance of particles and protein. Fluorescently labelled EVs were also detected after the same elution volume with an increase in the total fluorescence signal. By comparison, purification of labelling buffer which lacked LAC-CM-EVs resulted in no detectable enhancement in the fluorescence in the same fractions (Fig. 4b). Despite some particle loss following EVs labelling and purification (Fig. 4c) the reaction conditions were gentle. Furthermore, the particle size remained consistent after labelling (Fig. 4d) which indicated that aggregation of the labelled EVs was unlikely to be the mechanism which underpinned any particle loss. Instead, labelled EVs may have been retained in the qEV column or lost due to the post-column vesicle concentration step.^48^ Despite these particle losses, sufficient fluorescently labelled LAC-CM-EVs were recovered to enable further downstream applications and cell uptake studies.

### Uptake profile of LAC-CM-EVs by different human cardiac cells

Finally, to determine LAC-CM-EV uptake by specific cardiac recipient cells, we evaluated their uptake by the three major cell types comprising the heart including human cardiac fibroblasts (hCFib), human cardiac microvascular endothelial cells (hCMEC) and human iPSC-derived CMs (hCMs). The fluorescently labelled LAC-CM-EVs were detected using confocal microscopy with all cell types (green, Fig. 5a). High cell viability was observed in all the recipient cells after incubation with the LAC-CM-EVs (96.1% ± 4.1%, 95.9% ± 1.3%, 96.9% ± 1.1% for hCMEC, hCMs and hCFib, respectively; Fig. 5b). These high values were similar to the viability measured before LAC-CM-EV uptake in all cells tested (96.6% ± 0.7%, 96.3% ± 1.7%, 97.2% ± 2.7% for hCMEC, hCMs and hCFib, respectively), highlighting the overall low toxicity of the EVs. Interestingly, quantitative flow cytometry analysis of 488^+^ cells revealed significantly higher LAC-CM-EV uptake by the hCMEC (98.7% ± 1.1%), lower uptake (65% ± 13.3%) by the hCMs, while the lowest uptake was observed with hCFib (30.1% ± 10.1%, Fig. 5c). In the adult heart, the CMs are in close physical proximity to endothelial cells (ECs), and this enables efficient diffusion of oxygen and nutrients between the blood capillaries and the cardiac cells. This anatomical relationship also suggests possible crosstalk and information transfer between CMs and ECs. A growing body of evidence supports that CMs can modulate ECs function via soluble factors such as growth factors, hormones and genetic materials which further strengthen that a direct path of communication exists between these two cell types.^55^ In this context, EVs also appear to play an important role in cell-to-cell communication with important evidence now emerging to support the potential role of EVs of cardiac origin in the complex interplay between the CMs and ECs.^56^ Hence, we hypothesize that the observed differences in the uptake capacity may indicate a preferential uptake of LAC-CM-EVs by hCMEC due to the enhanced cellular communication of CMs in a low glucose environment with ECs. This hypothesis is supported by recent studies showing that EVs secreted from various cardiac cells strongly interact with ECs and have the capacity to modulate their behaviour and function. For example, EVs released from neonatal rat cardiomyocytes under glucose starvation were shown to transfer glycolytic enzymes and glucose transporters to ECs, thereby increasing their glucose uptake and glycolytic activity.^57^ Other studies showed that EVs derived from human cardiac progenitor cells (CPCs), MSCs and a cardiac myoblast cell line (H9c2) could stimulate the migration of ECs in an *in vitro* scratch wound assay as well as upregulating the expression of proangiogenic genes and promoting the formation of new functional vessels.^58–60^ Taken together, these studies suggest that EVs released by non-human CMs in low glucose environment augment intracellular communication with the ECs. Our findings, therefore, may shed new light on the fate of EVs released from human iPSC-CMs in the ischemic heart, their superior uptake by ECs and their potential involvement in cardiomyocyte to endothelial cell communication. Nevertheless, the mechanisms by which the LAC-CMs-EVs were internalized into the recipient cells and the exact biological processes involved remain to be determined. Overall, improved understanding of EV targeting and recipient cell uptake capacity may pave the way for new possibilities for the design of selective delivery approaches of therapeutic molecules. Moreover, the EVs can potentially be further engineered by manipulating their cargo for targeted delivery and enhanced cardiac repair. Future studies will be necessary to evaluate whether LAC-CM-EVs preferably interact with endothelial cells *in vivo* and potentially modulate their function in small and large animal models of cardiac injury. To conduct such studies significant scaling-up procedures would need to be developed in order to derive clinically relevant numbers of purified EVs from human iPSC-CMs. We expect that these studies could require considerably more hands-on time for cell culture and EV separation, to further investigate the clinical potential of purified EVs from human iPSC-CMs.

## Conclusions

EVs are attracting considerable interest in the cardiovascular field and have been shown to play an important role in improving cardiac function and promoting recovery after ischemic insult.^61^ However, one of the major challenges for the therapeutic translation of EVs is the introduction of unwanted contaminants by widely used recovery techniques.^7^ Here, we demonstrated a reproducible and standardisable EV isolation technique based on SEC that allowed efficient purification of intact EVs from homogenous population of human CMs. By tagging cardiac EVs with an amine reactive fluorescent dye we were further able to evaluate their interplay with the three major cell types comprising the heart, showing preferential uptake by human ECs. These findings represent a great promise for the future use of human iPSC-CM-EVs as cell-free therapeutic agents in cardiac repair.
